# Survival expectation after thrombosis and overt-myelofibrosis in essential thrombocythemia and prefibrotic myelofibrosis: a multistate model approach

**DOI:** 10.1038/s41408-023-00887-7

**Published:** 2023-07-28

**Authors:** Alessandra Carobbio, Alessandro Maria Vannucchi, Elisa Rumi, Valerio De Stefano, Alessandro Rambaldi, Giuseppe Carli, Maria Luigia Randi, Heinz Gisslinger, Francesco Passamonti, Juergen Thiele, Naseema Gangat, Ayalew Tefferi, Tiziano Barbui

**Affiliations:** 1 FROM Research Foundation, Papa Giovanni XXIII Hospital, Bergamo, Italy; 2 Center Research and Innovation of Myeloproliferative Neoplasms (CRIMM), Department of Experimental and Clinical Medicine, Azienda Ospedaliera Universitaria Careggi, University of Florence, Florence, Italy; 3 Department of Molecular Medicine, University of Pavia, Pavia, Italy; 4 Division of Hematology, Fondazione Istituto di Ricovero e Cura a Carattere Scientifico Policlinico San Matteo, Pavia, Italy; 5 Institute of Hematology, Catholic University, Rome, Italy; 6 Fondazione Policlinico ‘A. Gemelli’’ IRCCS, Rome, Italy; 7 Hematology and Bone Marrow Transplant Unit, ASST Papa Giovanni XXIII, Bergamo, Italy; 8 Department of Oncology and Hematology, University of Milan, Milan, Italy; 9 Division of Hematology, ‘‘S. Bortolo’’ Hospital, Vicenza, Italy; 10 Department of Medicine - DIMED, University of Padova, Padova, Italy; 11 Department of Internal Medicine I, Division of Hematology and Hemostaseology, Medical University of Vienna, Vienna, Austria; 12 Fondazione IRCCS Ca’ Granda Ospedale Maggiore Policlinico, Milan, Italy; Department of Oncology and Hemato-Oncology, University of Milan, Milan, Italy; 13 Institute of Pathology, University of Cologne, Cologne, Germany; 14 Hematology Division, Mayo Clinic, Rochester, MN USA

**Keywords:** Myeloproliferative disease, Epidemiology

Dear Editor

Ample evidence has been provided that accurate discrimination between essential thrombocythemia (ET) and early prefibrotic primary myelofibrosis (pre-PMF) has an impact not only on presenting laboratory data but also on complications, like thrombosis, progression to overt myelofibrosis (MF), transformation to blast phase (BP), and overall survival [1–8]. However, studies estimating the epidemiology of these critical events in the two entities have mainly focused on one isolated outcome at a time, without considering the entire spectrum of multiple intermediate disease states, possibly affecting probabilities and risk factors of the outcome of interest. This situation calls for a multistate model approach, a technique that allows a more in-depth insight into intermediate factors likely influencing the progressive transitioning from one status to another.

The aim of the present investigation was to estimate the probabilities that intermediate-state passages, including thrombosis, overt MF, and BP, impact the final absorbing state (death) in ET versus pre-PMF. To this purpose, retrospective data from two multicenter and well-documented studies [1, 9] were used: (i) ET patients (*n* = 791) from a multicenter international study of 891 cases, selected for the availability of complete disease history [1] and (ii) pre-PMF patients (*n* = 382) from four different Italian centers [9]. Both studies were approved by all institutional review boards or ethical committees of participating centers.

At the time of diagnosis, treatment-naïve ET and pre-PMF patients revealed different hematologic and clinical characteristics (Table S1). A parametric Markov multistate model [10] was applied to analyze data on survival considering intermediate states that are part of the natural history of ET and pre-PMF. The model included five states with ten possible transitions (Fig. S1): all patients begin in the initial state of diagnosis (ET, panel A, *n* = 791 or pre-PMF, panel B, *n* = 382) and then they could transit through the occurrence of an incident thrombotic event (Table S2) and/or the evolution to overt MF and/or BP (transient states) before death (absorbing state).

In ET, transition-1 from diagnosis to thrombosis included 101/791 patients (12.7%), but this status was transient in 21/101 patients that moved to death (21%) after a median time of 4.0 years (IQR: 1.6–6.4), 3/101 (6%) and in 1/101 (2%) to MF and BP, after a median time of 4.7 and 5.2 years, respectively. Remarkable was that in pre-PMF, the direct transition to thrombosis was found in 13.9%, a figure not different from ET (i.e., 12.7%). Conversely, pre-PMF substantially differed from ET for a higher rate of direct transition to overt MF or BP, that was 13 and 4% vs. 4 and 0.6%, respectively.

After 10 years, the state occupation probability of being event-free was 70 and 50% in ET and pre-PMF, respectively, and progressively decreased, particularly in pre-PMF (Fig. S2), due to earlier mortality, particularly for a greater probability of hematological evolutions. This trend was even more evident for death; regardless of the pathways through hematological evolutions, deaths were double in pre-PMF than ET, reaching 30, 60, and 80% vs. 15, 30, and 60% at 5, 10, and 20 years, respectively.

Probabilities to direct transition to thrombosis (*n* = 101 in ET and *n* = 53 in pre-PMF) and overt MF (*n* = 29 in ET and n = 51 in pre-PMF) are compared in Fig. 1. The trend of experiencing thrombosis directly after ET diagnosis showed to increase in the first 10 years (10%) and to decline subsequently (less than 5% at 30 years). On the contrary, in the first decade after diagnosis (<5%), the same probability grew slowly in pre-PMF while subsequently rose up to crossing the ET trend (8% after 30 years). Instead, the direct transitions from pre-PMF to overt-MF had an opposite trend: in the first 10 years, it reached a peak of 11%, while in ET, the trend was less pronounced, reaching a probability not exceeding 2.3% in the same period post-diagnosis.

**Fig. 1:**
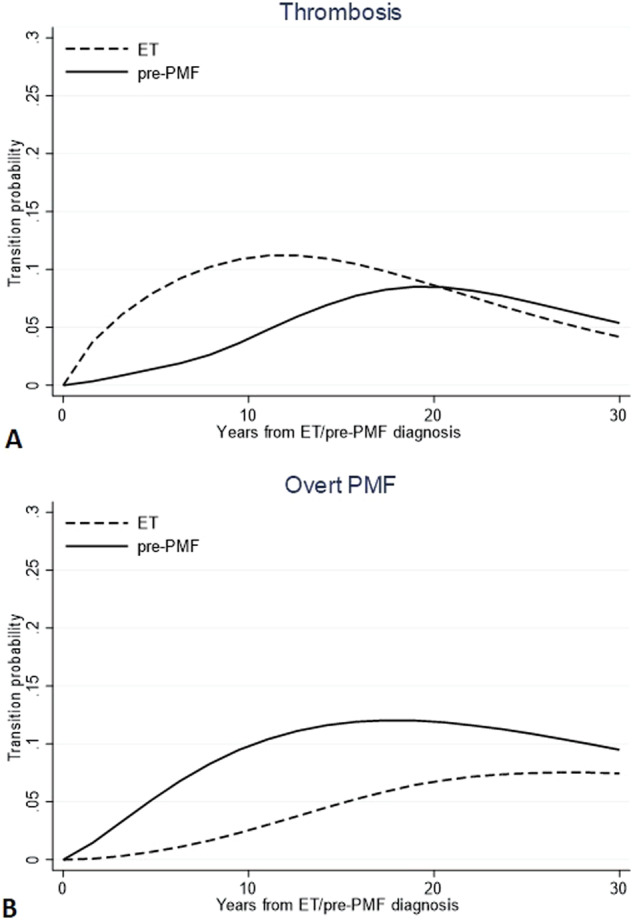
Direct transition probabilities to thrombosis and evolution in overt MF. Direct transition probabilities over time from diagnosis of ET or pre-PMF to thrombosis (**A**) and overt MF (**B**). Transition probabilities are defined as the probability of going from a given state to the next state in a Markov process. Direct transitions refer to all the 791 and 382 ET and pre-PMF patients, respectively, initially at risk; thus, they represent the probability that a patient can first experience thrombosis or evolve into overt MF.

The performance of the IPSET-thrombosis score [11] was tested in both ET and pre-PMF for the direct transition to thrombosis. In ET, considering the low-risk group as a reference, the intermediate and high-risk groups determined by IPSET-thrombosis were confirmed to predict the thrombotic risk (HR = 2.08, 95% CI = 1.28–3.37, *p* = 0.003 and HR = 3.13, 95% CI = 1.82–5.40, *p* < 0.001, respectively). In pre-PMF, the same model was unpowered to reach statistical significance in the intermediate-risk group (HR = 2.50, 95% CI = 0.87–7.21, *p* = 0.089), while it was in the high-risk category (HR = 3.93, 95% CI = 1.52–10.11, *p* = 0.005).

Concerning survival, most of the deaths in ET and pre-PMF occurred directly from diagnosis (Fig. 2). The intermediate events that most influenced death were thrombosis (25.3%) in ET and BP (23.8%) in pre-PMF. In comparison with ET, the probability of direct transition from diagnosis to death in patients with pre-PMF increased linearly over time (Fig. 2) and was twofold higher, reaching values of 15, 30, and 60% at 5, 10, and 20 years, respectively. Of note, the probability of death in ET patients with an intermediate thrombosis state maintained a fourfold higher value over time than the ones without thrombosis. As expected, the probabilities of death in MF or BP status were higher and occurred faster, and not substantially different in ET or pre-PMF.

**Fig. 2:**
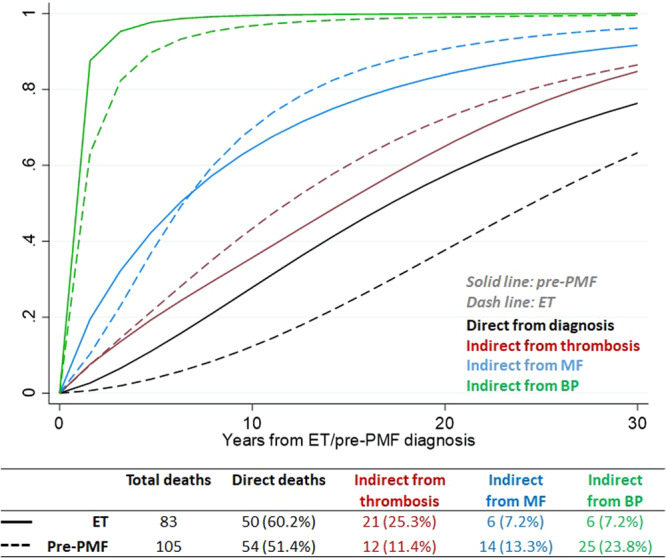
Transition probabilities to death in ET and pre-PMF. Comparison of the direct and indirect (via thrombosis, evolution in MF or BP) transition probabilities to death (absorbing state) over time from diagnosis of ET (dash lines) or pre-PMF (solid lines).

We confirmed the good performance of the IPSET-survival score [12] to differentiate the risk of direct mortality in ET (intermediate: HR = 4.38, 95% CI = 1.63–11.73, *p* = 0.003 and high-risk: HR = 20.17, 95% CI = 7.71–52.78, *p* < 0.001, compared to low-risk). The IPSET-survival score was equally well performing in pre-PMF (HR = 3.52, 95% CI = 1.22–10.12, *p* = 0.019 and HR = 13.37, 95% CI = 4.63–38.57, *p* < 0.001, for intermediate and high-risk groups, respectively, compared to low-risk). However, the discriminatory power of the IPSET-survival in ET was lower when the multistate model evaluated the mortality mediated by the thrombosis state; only high-risk patients were discriminated (HR = 19.27, 95% CI = 2.46–51.01, *p* = 0.005), whereas the intermediate-risk group was not significantly different from the low-risk one (HR = 3.84, 95% CI = 0.55–26.12, *p* = 0.194). Thus, in addition to the IPSET-survival risk factors (i.e., age ≥60 years, previous thrombosis, and white blood cells count ≥11 × 10^9^/L) we found that platelets count ≥1000 × 10^9^/L (HR = 5.74, 95% CI = 1.79–18.40, *p* = 0.003) and arterial vs. venous thrombosis in the follow-up (HR = 4.43, 95% CI = 1.04–18.91, *p* = 0.044) were independent predictors. The low number of deaths after thrombosis (12/105, 11%) in pre-PMF did not allow us to analyze the IPSET-survival performance in this transition.

The present multistate analysis, provides new insights for a better understanding of ET and pre-PMF disease processes. For example, in pre-PMF, the probability of thrombosis in the first decade was lower (<5%) due to a strong competitor represented by the evolution in MF (up to 11%). Consequently, occupation of thrombosis state in the first decade was lower in pre-PMF than in ET patients but became comparable in the last decades of observation (13 and 14% in ET and pre-PMF, respectively), supporting our previous cumulative estimates obtained with conventional methodology [1]. This notion might have practical implications to differentiate treatments during the course of the two entities by preferring antithrombotic prophylaxis according to IPSET thrombosis in ET that kept its discriminatory power also in this multiple competing adjustment analysis. In pre-PMF, therapy of first choice might be directed to prevent myelofibrosis evolution, provided agents able to do that are positively evaluated in appropriate clinical trials.

Regarding BP evolution, we highlight that the direct transition from the diagnosis was predominant in ET (*n* = 6/7, 86%), and it was modestly influenced by the pathway through thrombosis (*n* = 1/7, 14%). Unfortunately, we could not provide sufficient information on the role of cytoreductive therapy in these transitions due to the unreliable timing of drug administration.

Mortality prediction in ET was the topic addressed in a previous study [12]. On the basis of the hazard ratio estimates from Cox regression models, the IPSET-survival model was constructed, and 867 ET patients were allocated into three risk categories with significantly different survival [12]. In the present analysis, in a selected group of patients (*n* = 791) from the same database, we re-evaluated the risk factors of death considering the possible influence of the intermediate states that occurred before death, and confirmed the performance of the IPSET-survival scoring system for the prediction of direct mortality. However, we also found that the effect on mortality exerted by the intermediate thrombosis state was not negligible (accounting for 25% of deaths) and fourfold higher than in patients without incident thrombosis. Whether the reduction of vascular complications may impact survival remains to be demonstrated in appropriate prospective studies. Furthermore, we found two additional independent predictors of mortality in thrombosis-mediated transition, namely platelet count >1000 × 10^9^/L (HR = 5.74, 95% CI = 1.79–18.40, *p* = 0.003), in line with a previous observation [13], and the incident arterial vs. venous thrombosis (HR = 4.43, 95% CI = 1.04–18.91, *p* = 0.044).

Limitations of this study concern its retrospective design and a possible bias related to the reporting accuracy of events, in terms of completeness and timing. In addition, since in these databases, the administration times of the cytoreductive drugs (hydroxyurea in absolute prevalence) were not well specified, we could not reliably evaluate the influence of the pharmacological cytoreduction on the post-diagnosis events. Furthermore, given that current results were obtained in the same ET database used for IPSET scores, a possible "self" confirmation bias could not be excluded. However, our aim was not to confirm the overall performance of the two scores, but to evaluate whether the transition from one state to another could have affected the overall survival or the cumulative incidence of thrombosis in a different way.

Strengths of the study are the relatively large number of patients for rare diseases such as ET and pre-PMF and the clinical and hematological diagnostic accuracy of the two entities and outcomes.

In conclusion, this multistate analysis provides novel information on the temporal probability of intermediate critical events occurring in ET and pre-PMF, and their impact on mortality. This knowledge might inform clinical practice and could also make more feasible the design of clinical trials.

## Supplementary information

Supplemental material
