# Supplementary material for: Survival expectation after thrombosis and overt-myelofibrosis in essential thrombocythemia and prefibrotic myelofibrosis: a multistate model approach
Source: Blood Cancer J. 2023 Jul 28;13(1):115. doi: 10.1038/s41408-023-00887-7 (PMC10382585; doi:10.1038/s41408-023-00887-7)
Supplement: Supplementary file 1 — Supplemental material [file 41408_2023_887_MOESM1_ESM.pdf]

Supplementary materials

Table S1. Clinical characteristics of 791 ET and 382 pre-PMF patients.

**Clinical features**	**ET N=791**	**Pre-PMF N=382**	**p-value**
Male/Female, n (%)	328/463 (41.5/58.5)	195/187 (51.0/49.0)	0.002
Age, years, median (IQR)<br>>60 years, n (%)	55.3 (42.8-67.4)<br>330 (41.7)	57.6 (43.1-69.0)<br>170 (44.5)	0.11<br>0.38
Hemoglobin, g/dL, median (IQR)	14.1 (13.3-15.0)	13.5 (12.1-14.7)	<.001
Platelets, $x10^9$/L, median (IQR)	779 (644-970)	700 (493-966)	<.001
Leukocytes, $x10^9$/L, median (IQR)	8.6 (7.2-10.5)	10.0 (7.8-12.8)	<.001
*JAK2*V617F, n (%)	362/596 (60.7)	246 (64.4)	0.17
Fibrosis grade, n (%)<br>0<br>1	 768 (97.1)<br>23 (2.9)	 161 (42.1)<br>221 (57.9)	<.001
Palpable splenomegaly, n (%)	117 (14.8)	176 (46.1)	<.001
Lactate dehydrogenase > 1.5 times the upper limit of normal, n (%)	21/381 (5.5)	119/298 (39.9)	<.001
Circulating CD34 cells, median (IQR)	2.0 (0.0-4.0) n=228	7.4 (3.0-21.6) n=211	<.001
Previous thrombosis, n (%)<br>Arterial<br>Venous	164 (20.7)<br>119 (15.0)<br>55 (7.0)	65 (17.0)<br>35 (9.2)<br>31 (8.1)	0.13<br>0.002<br>0.47
Cardiovascular risk factors, n (%)	190 (24.0)	161/360 (44.7)	<.001

Legend: Cardiovascular risk factors –at least one among smoking, arterial hypertension and diabetes

Table S2. Types of thrombosis during follow-up

	**ET (n=791)**	**Pre-PMF (n=382)**
**Total thrombosis**	101 (12.8%)	53 (13.9%)
**Arterial**	75 (9.5%)	30 (7.9%)
AMI	*16 (2.0%)*	*9 (2.4%)*
Stroke/TIA	*44 (5.6%)*	*14 (3.7%)*
PAT	*15 (1.9%)*	*7 (1.8%)*
**Venous**	35 (4.4%)	28 (7.3%)
DVT/PE		*12 (3.1%)*
SVT		*8 (2.1%)*
Abdominal/Budd-Chiari		*8 (2.1%)*

Legend. Nine and 5 patients have both arterial and venous thrombosis in ET and pre-PMF, respectively.

Figure S1. Five-states models in ET and pre-PMF

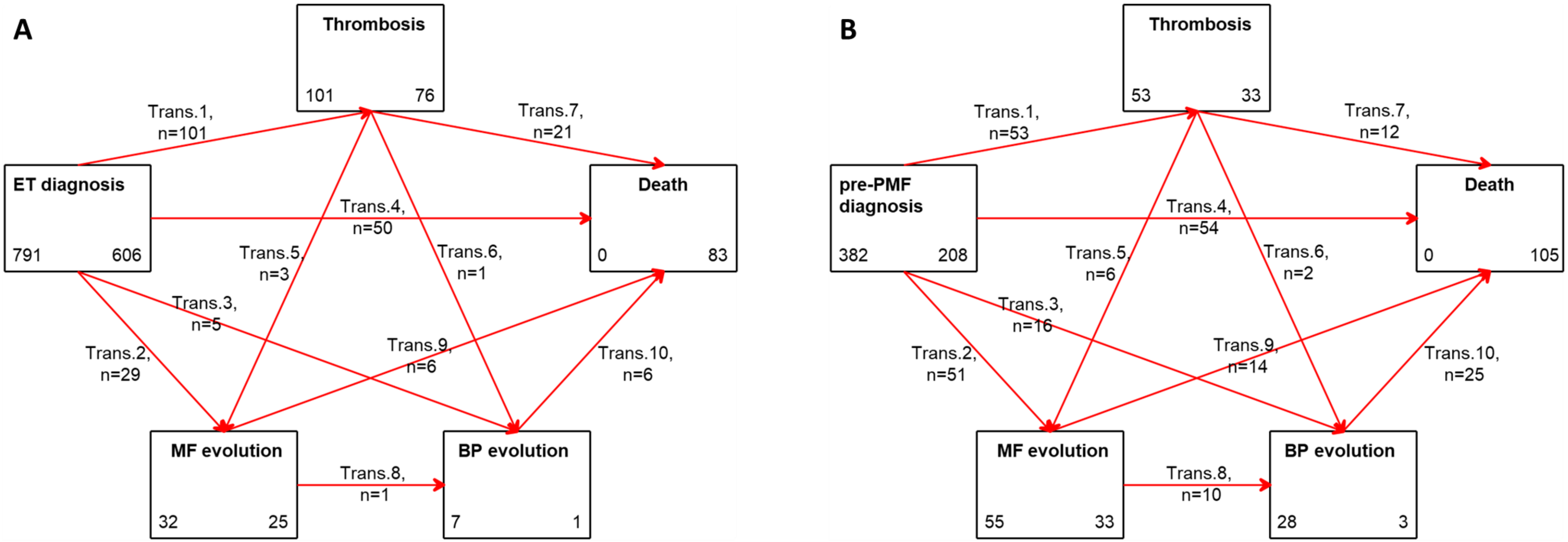

Legend. The five stages (i.e., ET/pre-PMF diagnosis, thrombosis, MF evolution, BP evolution and death) are graphed by boxes. In each box the corresponding number of patients is given at presentation (left corner) and ending (right corner) regarding their state. Across the indicated states, 10 possible transitions are graphed by arrows, as following: from ET/pre-PMF diagnosis to thrombosis (trans.1), MF evolution (trans.2), BP evolution (trans.3) or death (trans.4); from thrombosis to MF (trans.5), BP (trans.6) or death (trans.7); from MF to BP (trans.8) or death (trans.9); from BP to death (trans.10).

Figure S2. Stacked state occupation probabilities in ET and pre-PMF

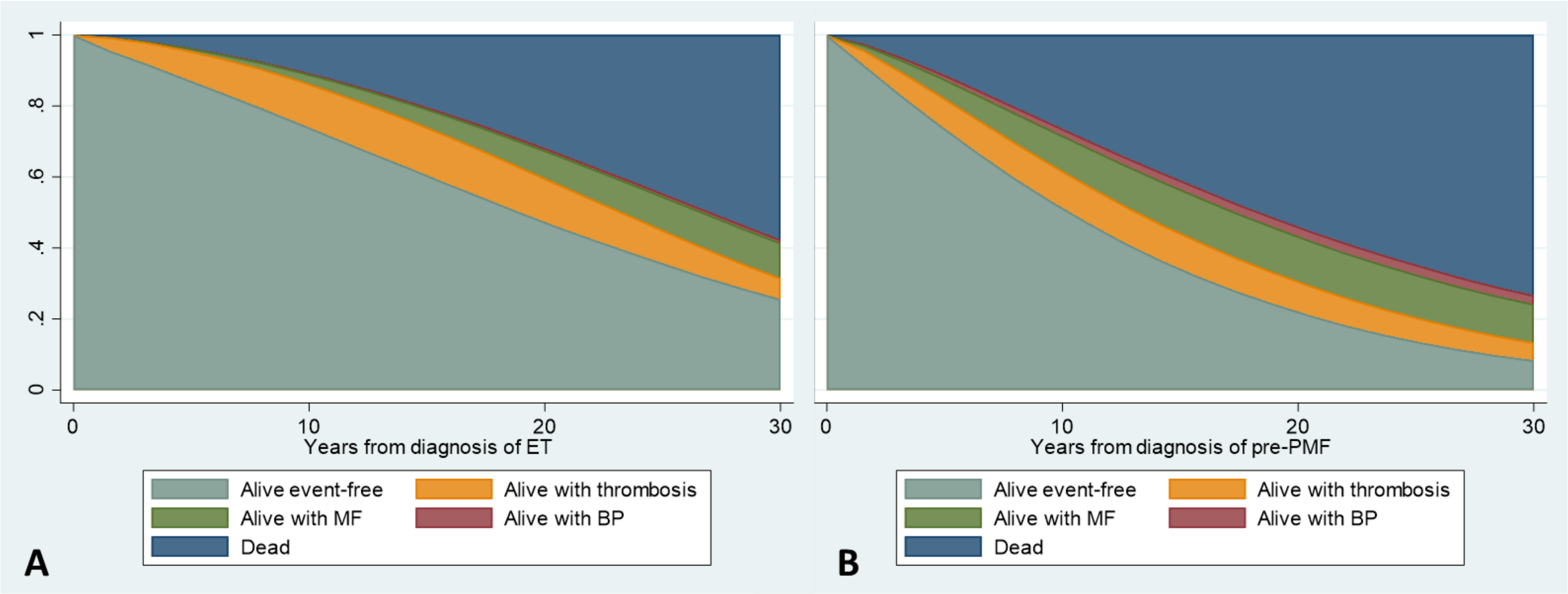

Legend. Stacked plot of state occupation probabilities of being alive free-from thrombosis, evolution into overt MF, BP, or having died as a function of time since diagnosis of ET (panel A) and pre-PMF (panel B). The figure shows the state occupation probabilities, which are the probability of being in a state at a certain time. The stacked presentation allows to compare the four different probabilities simultaneously. At the time 0 (diagnosis), the probability of being alive and free-from events is 100%. During the course of the diseases, this probability gradually decreases in favor of other states occupation.
